# Sources of Variability in Platelet Accumulation on Type 1 Fibrillar Collagen in Microfluidic Flow Assays

**DOI:** 10.1371/journal.pone.0054680

**Published:** 2013-01-23

**Authors:** Keith B. Neeves, Abimbola A. Onasoga, Ryan R. Hansen, Jessica J. Lilly, Diana Venckunaite, Meghan B. Sumner, Andrew T. Irish, Gary Brodsky, Marilyn J. Manco-Johnson, Jorge A. Di Paola

**Affiliations:** 1 Department of Chemical and Biological Engineering, Colorado School of Mines, Golden, Colorado, United States of America; 2 Department of Pediatrics, Hemophilia and Thrombosis Center, University of Colorado Denver, Aurora, Colorado, United States of America; Institut National de la Santé et de la Recherche Médicale, France

## Abstract

Microfluidic flow assays (MFA) that measure shear dependent platelet function have potential clinical applications in the diagnosis and treatment of bleeding and thrombotic disorders. As a step towards clinical application, the objective of this study was to measure how phenotypic and genetic factors, as well as experimental conditions, affect the variability of platelet accumulation on type 1 collagen within a MFA. Whole blood was perfused over type 1 fibrillar collagen at wall shear rates of 150, 300, 750 and 1500 s^−1^ through four independent channels with a height of 50 µm and a width of 500 µm. The accumulation of platelets was characterized by the lag time to 1% platelet surface coverage (Lag_T_), the rate of platelet accumulation (V_PLT_), and platelet surface coverage (SC). A cohort of normal donors was tested and the results were correlated to plasma von Willebrand factor (VWF) levels, platelet count, hematocrit, sex, and collagen receptors genotypes. VWF levels were the strongest determinant of platelet accumulation. VWF levels were positively correlated to V_PLT_ and SC at all wall shear rates. A longer Lag_T_ for platelet accumulation at arterial shear rates compared to venous shear rates was attributed to the time required for plasma proteins to adsorb to collagen. There was no association between platelet accumulation and hematocrit or platelet count. Individuals with the AG genotype of the *GP6* gene had lower platelet accumulation than individuals with the AA genotype at 150 s^−1^ and 300 s^−1^. Recalcified blood collected into sodium citrate and corn trypsin inhibitor (CTI) resulted in diminished platelet accumulation compared to CTI alone, suggesting that citrate irreversibly diminishes platelet function. This study the largest association study of MFA in healthy donors (n = 104) and will likely set up the basis for the determination of the normal range of platelet responses in this type of assay.

## Introduction

The central role of shear stress in thrombus formation is well documented. Platelets can adhere to fibrinogen and collagen at venous shear stresses, but von Willebrand factor (VWF) is necessary to promote rolling prior to firm adhesion at arterial shear stresses [1], [2]. The binding of VWF to collagen is also shear stress dependent where high shear stress exposes the A1 domain, which can then substitute for the collagen binding site in the A3 domain [3]. Recent studies have shown that VWF multimer size is regulated by shear stress where a threshold shear stress gradient exposes the A2 domain allowing cleavage by ADAMTS13 [4]. Furthermore, the rate of transport of coagulation zymogens and enzymes to and from a clot depend on shear rate. For example, fibrin formation is inhibited at high shear rates because fibrin monomers and thrombin are washed out before fibrin fibers can form [5]. Despite these numerous shear stress and shear rate dependent mechanisms, there is no accepted clinical method to evaluate thrombus formation under physiological shear stresses.

Flow assays continue to be an indispensible research tool that best recreate the hemodynamic conditions of the vasculature. However, the high volume (10–100 mL) requirements and low throughput of annular and parallel plate flow chambers make them prohibitive for a clinical assay. In the last few years, there have been several reported methods that use a combination of microfluidic channels and micropatterning of prothrombotic proteins to address these issues [6], [7]. Microfluidic channels with dimensions of 10–100 µm reduce the amount of whole blood required to 0.1–1 mL. Fabricating multiple channels as part of a single device allows for higher throughput to simultaneously measure platelet function over a range of shear stresses and to perform dose-response experiments for antiplatelet agents [8]–[10]. Given these advances and the commercialization of microfluidic platforms for cell adhesion assays [11], [12], it is timely to explore their translation into a clinical assay.

If flow assays are to become a clinical tool, the normal response must be quantified. This is important because without characterizing the normal range within the assays, we will not be able to discriminate between normal and abnormal responses. The variability in platelet function within in the normal population is significant. This variability stems from several genotypic and phenotypic differences between individuals [13], [14]. The objective of this study was to measure how some of the previously identified phenotypic and genetic factors known to affect platelet function, as well as certain experimental conditions (collagen surface density, anticoagulant, assay duration), effect the variability in platelet accumulation on type 1 fibrillar collagen at venous and arterial shear rates in a microfluidic flow assay (MFA) [15]–[17]. We evaluated the combined role of hematocrit, platelet count, sex, VWF levels and collagen receptor genotypes on platelet accumulation under flow in 50 healthy individuals. Neither hematocrit nor platelet count within the normal ranges were found to affect platelet accumulation. We found VWF plasma levels, and *GP6* genotype to be significant factors in platelet function on type 1 collagen under flow. A longer lag time for platelet accumulation at arterial shear rates compared to venous shear rates was attributed the need for adsorption of certain plasma proteins, presumably VWF, prior to platelet adhesion.

## Materials and Methods

### Materials

Equine tendon fibrillar type 1 collagen was purchased from Chrono-log Corp (Havertown, PA). PE/Cy5 labeled mouse antihuman CD41a (HIP8 monoclonal antibody) was from BD Pharmingen (San Jose, CA). Gluteraldehyde (25%, EM Grade) was purchased from Polysciences, Inc. (Warrington, PA). Phosphate buffered saline was from Gibco (Grand Island, NY). Bovine serum albumin and all other chemicals were purchased from Sigma-Aldrich (St. Louis, MO). HEPES buffered saline (HBS) was made in-house.

### Recruitment of Human Subjects

Healthy volunteers were recruited at the Hemophilia and Thrombosis Center of the University of Colorado Anschutz Medical Campus in accordance with the Declaration of Helsinki. The study received institutional review board approval from the University of Colorado IRB, and written informed consent was obtained for all participants. Subjects were not included if they had: a) consumed aspirin within 10 days of blood draw; b) ingested non steroidal anti-inflammatories (NSAIDS) within 4 days prior to phlebotomy; c) ingested alcohol within 24 hours prior to phlebotomy; d) reported feeling ill within 7 days prior to phlebotomy; e) reported a first-degree family history of bleeding disorders or stroke, heart attacks or deep vein thrombosis before the age of 50.

### Blood Collection

Human whole blood was collected by venipuncture into 3.2% sodium citrate and 50 µg/mL corn trypsin inhibitor vacutainers or into 50 µg/mL corn trypsin inhibitor vacutainers (Haematologic Technologies Inc, Essex Junction, VT) after the first 8 mL of blood were discarded. The whole blood was incubated with a non-function blocking anti-CD41 antibody for 10 min and then recalcified to 5 mM CaCl_2_ immediately prior to introduction of the blood into the device. Blood was used between 30–60 min after phlebotomy. Complete blood counts (CBC) were obtained for all recruited individuals.

### Collagen Patterning

**Figure 1:**
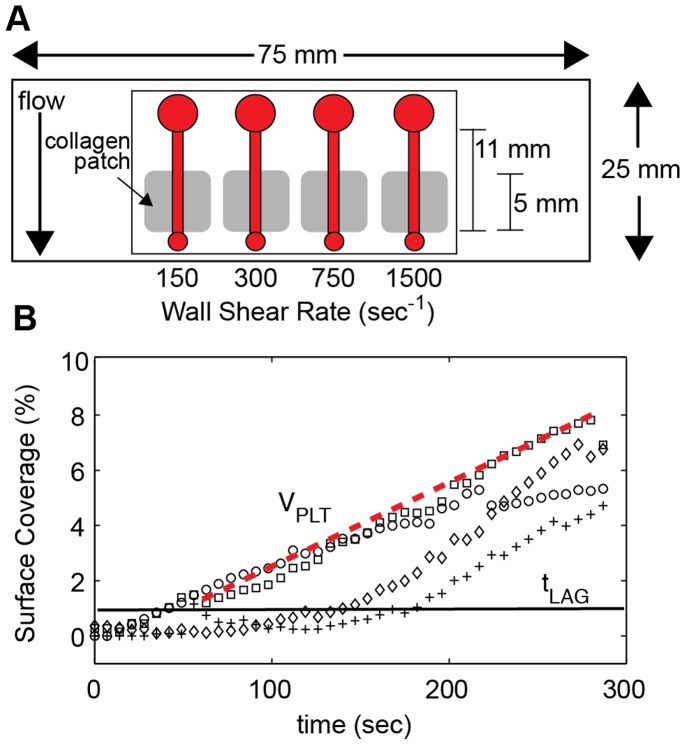
Microfluidic flow assay and quantification of platelet accumulation. A. Schematic of the microfluidic flow assay. Four channels (h = 50 µm, w = 500 µm) were placed over a patch of type 1 collagen. Blood was pipetted in an inlet well (large circle) and withdrawn through the outlet (small circle) at a constant flow rate to achieve the desired wall shear rate. Platelet accumulation was monitored at the upstream edge of the collagen patch by epifluorescence microscopy. B. Platelet surface coverage was measured over the course of 5 min at 150 s^−1^ (○), 300 s^−1^ (□), 750 s^−1^ (⋄) and 1500 s^−1^ (+). In addition to the final platelet surface coverage (SC), the lag time (Lag_T_, black line) and the accumulation velocity (V_PLT_, red dotted line) were calculated from each curve. Lag time was defined as the time to 1% surface coverage. Accumulation velocity was defined as the slope of line SC versus time from t_LAG_ to 5 min.

Glass slides were cleaned in 1∶1 solution of methanol:hydrochloric acid (37 N) for one hour, thoroughly rinsed in deionized water, and then dried with compressed air. Slides were coupled to a 16-well incubation chamber (FAST Frame, Whatman, Piscataway, NJ) and loaded into holder (Chip Clip, Whatman). The collagen was diluted to 5, 10 50, 100, 200, 500 or 1000 µg/mL in a 5% glucose solution; a 100 µL was pipetted into four of the wells, and then allowed to adsorb to the glass slides for one hour at room temperature. Following incubation, the wells were rinsed twice with 5% glucose, and the slide was removed from the holder, thoroughly rinsed with deionized water, and gently dried with compressed air. The result of this procedure was four 5 mm x 5 mm patches of collagen spaced 5 mm (edge-to-edge) apart (Fig. 1A). Following collagen patterning, the slide was blocked with 1 mg/mL bovine serum albumin (BSA) for one hour at room temperature.

### Microfluidic Flow Assays

Microfluidic flow chambers were fabricated in polydimethylsiloxane (PDMS) from silicon masters using standard soft lithography methods with a similar design as previously reported [7]. The master was made using deep reactive ion etching, which yields channel heights within 3% of each other across a four inch silcon wafer. Each device consisted of four independent channels with a width of 500 µm and height of 50 µm. Devices were blocked in 1 mg/mL BSA for 1 hr at room temperature. The devices were aligned with the collagen patches and reversibly sealed to the slide using vacuum assisted bonding as previously described [7]. The channels were first filled with HBS to check for any leaks and to remove any air bubbles. Recalcified whole blood was pipetted into the inlet chamber of the four channels and then was simultaneously withdrawn through at wall shear rates of 150, 300, 750, and 1500 s^−1^ for 5 min. Wall shear rate was related to volumetric flow rate through the following expressions:
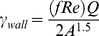
(1)

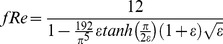
(2)where γ_wall_ is the wall shear rate, *f* is the friction factor, *Re* is the Reynolds number, *Q* is the volumetric flow rate, *A* is the channel cross sectional area, and ε is the channel aspect ratio (width/height). All experiments were performed at room temperature. In some experiments, autologous plasma from a donor was perfused over the surface for 10 min prior to introduction of whole blood to measure the adsorption of VWF to fibrillar collagen. The four different wall shear rates were achieved by attaching the outlet of each channel to a different sized glass syringes (50, 100, 250, 500 µL; 1700 Series Gastight Syringes, Hamilton Co, Reno, NV). The differences in the syringe diameters yield a ratio of flow rates of 1∶2∶5∶10 for the 50∶100∶250∶500 µL syringes. The syringes were placed in a single syringe pump (PHD2000, Harvard Apparatus), and all four channels were run simultaneously.

### Image Acquisition and Analysis

The accumulation of platelets was monitored in each channel at the upstream edge of the collagen patch using an inverted fluorescence (IX81, Olympus, Center Valley, PA) with a 20X objective (NA 0.45) equipped with a motorized stage (Proscan, Prior Scientific, Rockland, MA) and 16-bit CCD camera (Orca-ER, Hamamatsu). Image capture and stage movement was controlled with Slidebook 5.0 software (Intelligent Imaging Innovations, Denver, CO). An image was captured in each channel every 7 sec over the duration of the experiment. After 5 min, the channel was rinsed with autologous platelet poor plasma (PPP) for 2 min, and then rinsed with a 2.5% gluteraldehyde solution for 2 min to fix the platelet aggregate. Finally, the slide was immersed in 2.5% gluteraldehyde for 1 hr before being coverslipped. During the plasma rinse, another set of images was captured at the same position as the real-time images and at positions 1 mm and 2 mm downstream from the leading edge of the collagen spot. Images were exported as 8-bit TIFF for analysis.

Image analysis was performed using custom MATLAB (Mathworks, Natick, MA) scripts for both the transient platelet accumulation and the end-point images. This scripts are available on the MATLAB File Exchange website (www.mathworks.com/matlabcentral/fileexchange/) as Files #36820 and #36821. One script copies the contents from a source drive (DVD) to the hard drive (#36821). The second script converts RGB TIFFs into grayscale images, thresholds them based on the triangle algorithm [18], [19], removes any isolated groups of pixels less than the area of a single platelet, and then calculates the area fraction of platelets for each frame (#36820). For each set of images, three parameters were measured; (1) a lag time (Lag_T_) defined as the time when >1% of the surface was covered with platelets, (2) a platelet accumulation velocity (V_Plt_) defined as the slope of platelet area fraction as a function of time from Lag_T_ until the end of the experiment (t = 5 min), and (3) the percent surface coverage (SC) at the end of the experiment (Fig. 1B). Lag_T_ and V_Plt_ were calculated from the transient images taken during the experiment. The linear fit was performed using the *robustfit* algorithm in MATLAB.

### Plasma Von Willebrand Factor Levels

VWF:Ag was measured for all recruited individuals who provided a blood sample. VWF:Ag was measured on standard ELISA plates coated with a combination of two monoclonal antibodies as previously described [20]. Briefly, plasma samples were plated into duplicate wells for three different dilutions per sample. Captured VWF was detected with polyclonal rabbit antibody with an enzyme conjugate reaction. Agreement between dilutions was evaluated as a measure of quality.

### *ITGA2*, *GP6* and *GP1BA* Genotyping

We designed primers covering (rs1126643, *ITGA2*; rs1613662, *GP6*; rs6065, *GP1BA*) from genomic sequence per the UCSC genome browser (http://genome.ucsc.edu/) using Primer3 (available upon request). PCR was performed on genomic DNA and Sanger sequencing was performed using BigDye V3.1 on an ABI3730xl. Analysis was conducted using Sequencher V4.9.

### Statistical Analysis

All statistical analysis was performed using the Statistics Toolbox in MATLAB. The Mann-Whitney U-test was used to determine differences between pairs of categorical data. Kruskal-Wallis ANOVA was used to determine differences between groups, followed by a post hoc Tukey’s honestly significant difference test to determine differences between pairs. Two-way ANOVA was used to measure interactions between parameters. The Spearman correlation coefficient was calculated for continuous variables. All data is presented as the mean ± standard error unless otherwise noted.

## Results

Whole blood from 104 individual donors was tested in the MFA (Table 1). Of these donors, 54 were used to explore the effect of experimental conditions (collagen surface density, anticoagulant, time) and to quantify intra-individual variability, and 50 were used to characterize inter-individual variations. The device used in this study consisted of four independent channels with a height of 50 µm and a width of 500 µm (Fig. 1A). These channel dimensions were chosen based on previous work that showed that this channel aspect ratio (10∶1 width:height) yields a blunted shear stress profile that results in uniform platelet deposition [21]. Whole blood behaves as a Newtonian fluid in channels greater than 50 µm and shear rates greater than 100 s^−1^ [22]. Whole blood was perfused through the four channels at 150, 300, 750, and 1500 s^−1^. Platelet accumulation was characterized using three metrics; (1) platelet surface coverage (SC) at the end of the assay, (2) lag time to a SC of 1% (Lag_T_), and (3) the rate of platelet accumulation from Lag_T_ to the end of the assay (V_PLT_) (Fig. 1B). In some samples, the platelet accumulation did not reach a SC of 1%. For these samples, only the SC was included in the data analysis.

**Table 1:** Characteristics of the cohort of donors.

Total number of donors		104
Women		60 (58%)
Oral Contraception		13 (21% of women)
Age, mean ± stdev (range)		32.5±11.0 (21–74)
Hematocrit, mean ± stdev (range)		
	Combined	43.9±4.8 (27.0–54.1)
	Women	41.5±4.2 (27.0–46.9)
	Men	47.8±2.9 (44.0–54.1)
Platelet count (plt/µL), mean ± stdev (range)		
	Combined	311,000±56,000 (211,000–503,000)
	Women	321,000±64,000 (211,000–503,000)
	Men	291,000±30,000 (255,000–370,000)
Plasma VWF (IU/dL), mean ± stdev (range)		
	Combined	87.9±35.1 (26.3–178.2)
	Women	97.3±30.4 (38.5–178.2)
	Men	73.4±30.1 (26.3–151.4)

### Sensitivity to Collagen Surface Density

**Figure 2:**
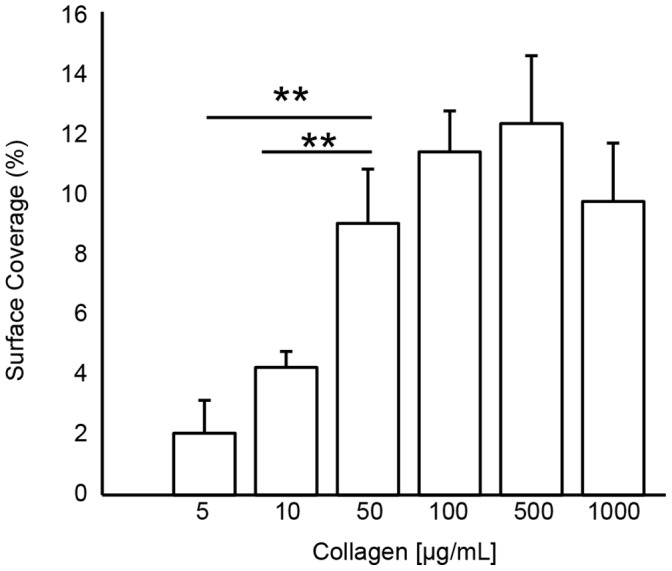
Sensitivity of platelet accumulation to collagen surface density. Type 1 fibrillar collagen was adsorbed to clean glass slides at solution concentrations of 5–1000 µg/mL. Whole blood was perfused over the collagen substrates at 300 s^−1^ and platelet accumulation was measured by fractional surface coverage. There was significantly lower platelet accumulation on 5 µg/mL and 10 µg/mL surfaces than on surfaces prepared from solutions of greater than 50 µg/mL. Lines with ** denotes a p<0.01 for the Mann-Whitney U-test.

Type 1 fibrillar collagen was adsorbed to clean glass from solutions of 5, 10, 50, 100, 200, 500 or 1000 µg/mL in order to measure the sensitivity of platelet accumulation to collagen surface concentration (Fig. 2). Whole blood was perfused over each surface at 300 s^−1^. Platelet accumulation as measured by SC was significantly lower (p<0.01) on the 5–10 µg/mL substrates than the higher collagen concentrations (n = 21). There was no statistical difference in SC over the range of 50–1000 µg/ml, suggesting that these surface concentrations of collagen exceed the surface concentration of collagen receptors on platelets. Therefore, differences between donors can be attributed to composition of plasma proteins such as VWF and collagen and VWF receptor density. We chose 100 µg/mL for all subsequent experiments because this a common concentration used for type 1 fibrillar collagen in flow assay studies [16].

### MFA Reproducibility

To quantify the reproducibility of the MFA, we tested five donors on four separate days over a two-week interval (Table 2). Phlebotomy was performed at the same time of day for each draw. The average coefficient of variation in SC was less than 0.15 at 150, 300, and 750 s^−1^, but larger at 1500 s^−1^ (0.45). We attribute the high CV for 1500 s^−1^ to the relatively low levels of platelet accumulation at this shear rate, rather than as an indication of a systematic source of variability within the assay. Donor 3 had very low binding, including insignificant platelet adhesion at 750 s^−1^, compared to the other donors. However, this behavior was reproducible for each test and indicative of the low binder group observed in the larger cohort (described in the next section). While the intra-donor variability is low, the inter-donor variability is quite high as indicated by the large standard deviation in SC between the five donors. A large cohort was recruited to identify the source of this variability.

**Table 2:** MFA intra-assay variation.

		Surface Coverage (%)											
Donor	Gender	150 s^−1^			300 s^−1^			750 s^−1^			1500 s^−1^		
		AVG	SD	CV	AVG	SD	CV	AVG	SD	CV	AVG	SD	CV
1	M	14.6	1.6	0.11	15.1	2.9	0.19	1.2	0.4	0.32	1.2	0.6	0.55
2	F	15.3	1.0	0.07	12.6	1.1	0.09	6.5	1.1	0.17	2.1	1.4	0.69
3	M	1.1	0.3	0.22	0.7	0.1	0.14	0.0	0.0	0.0	0.0	0.0	0.0
4	F	15.2	1.5	0.10	19.2	2.9	0.15	9.5	0.5	0.05	6.9	0.6	0.09
5	F	12.2	1.6	0.13	18.6	2.9	0.15	8.0	0.5	0.06	5.4	0.06	0.12
**Avg. SC**		**11.6±6.4**			**13.0±9.2**			**4.9±5.8**			**3.2±3.7**		
**Avg CV**		**0.12±0.06**			**0.15±0.11**			**0.15±0.11**			**0.45±0.33**		

Platelet percent surface coverage (SC) for five donors repeated four times. The average (AVG), standard deviation (SD), and coefficient of variability (CV) are reported for each donor at each wall shear rate. The average surface coverage and average coefficient of variation across all five donors is reported in the bottom two rows.

### Characteristics of Platelet Accumulation in the MFA in a Large Cohort of Normal Donors

**Figure 3:**
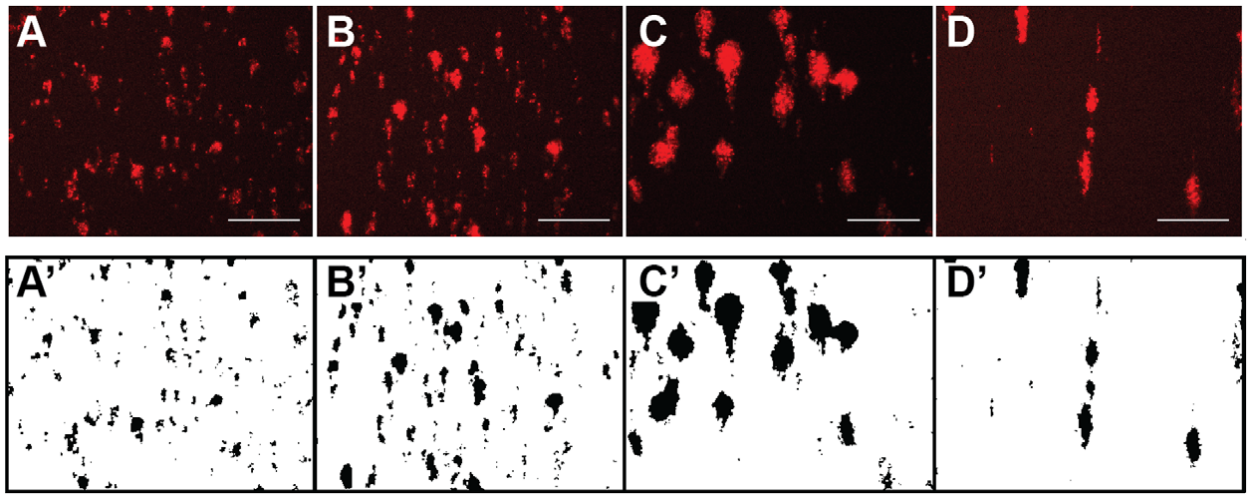
Platelet accumulation as function of shear rate. Platelets were labeled with a PE/Cy5 labeled mouse antihuman CD41a antibody and their accumulation was measured over the course of a 5 min flow assay. The top row shows the raw images and the bottom row shows the binary images following image processing at wall shear rates of 150 s^−1^ (A, A’), 300 s^−1^ (B, B’), 750 s^−1^ (C, C’) and 1500 s^−1^ (D, D’). Scale bar = 100 µm.

**Figure 4:**
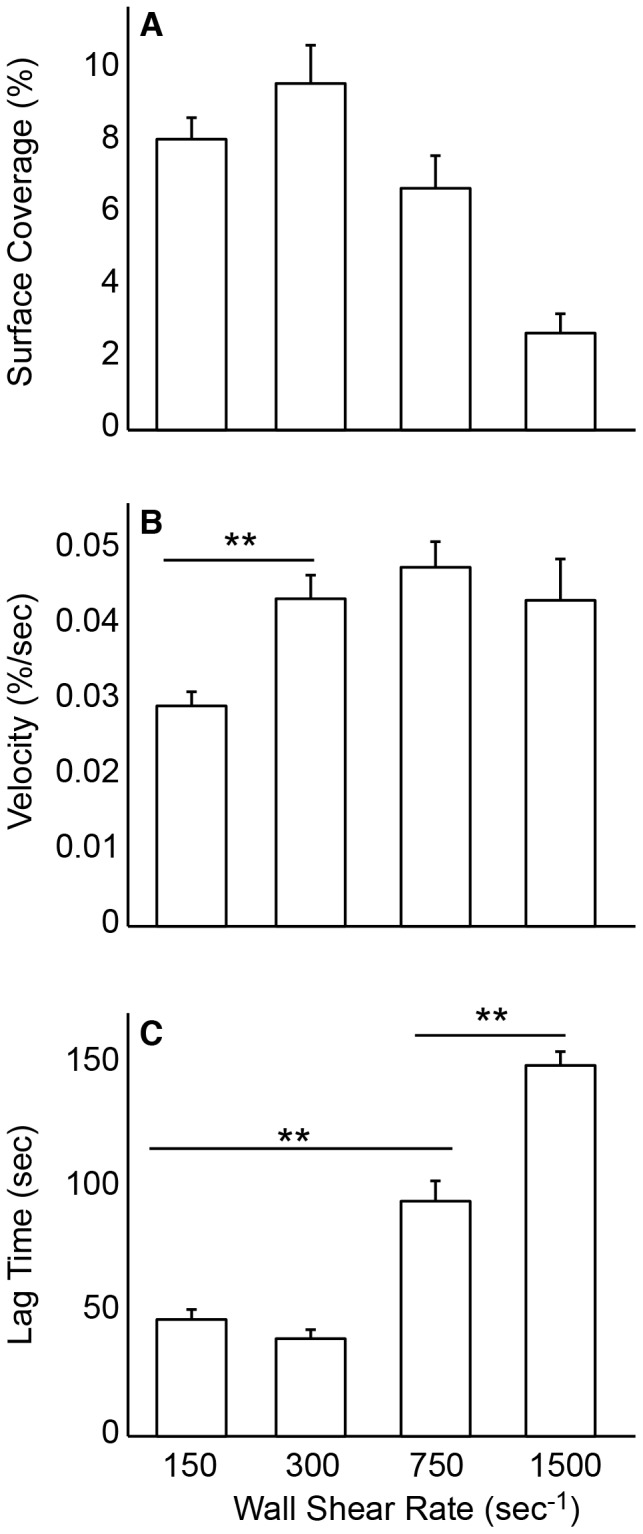
Surface coverage, rate and lag time of platelet accumulation as a function of wall shear rate. Recalcified citrated whole blood was perfused over type I collagen for 5 min and platelet accumulation was monitored as a function of wall shear rate and time (n = 50). Platelet accumulation was characterized by (A) percent surface coverage (SC) after 5 min, (B) the rate of platelet accumulation expressed as percent surface coverage per second (V_PLT_), and (C) the lag time to 1% surface coverage (Lag_T_). Lines with ** denotes a p<0.01 for the Mann-Whitney U-test.

Fifty normal donors were recruited and their platelet accumulation on type 1 collagen (100 µg/mL) was measured at 150, 300, 750 and 1500 s^−1^. Fig. 3 shows representative images before and after image processing at the end of a 5 min assay. Platelet SC peaked at 300 s^−1^ and was lowest at 1500 s^−1^ (Fig. 4A). The rate of platelet accumulation (V_PLT_) was lowest at 150 s^−1^ and different (p<0.01) than the other three shear rates (Fig. 4B). There was no difference in V_PLT_ between the higher three shear rates. The lag time (Lag_T_) was similar at 150 s^−1^ and 300 s^−1^, and significantly higher (p<0.01) at 750 s^−1^ and 1500 s^−1^ (Fig. 4C). The differences Lag_T_ between the low and high shear rates are associated with the time required for a significant amount of VWF to bind to the collagen (see *The lag time for platelet accumulation at high shear rates is due to adsorption of plasma proteins* below).

### Platelet Accumulation Correlates to VWF Plasma Levels

**Figure 5:**
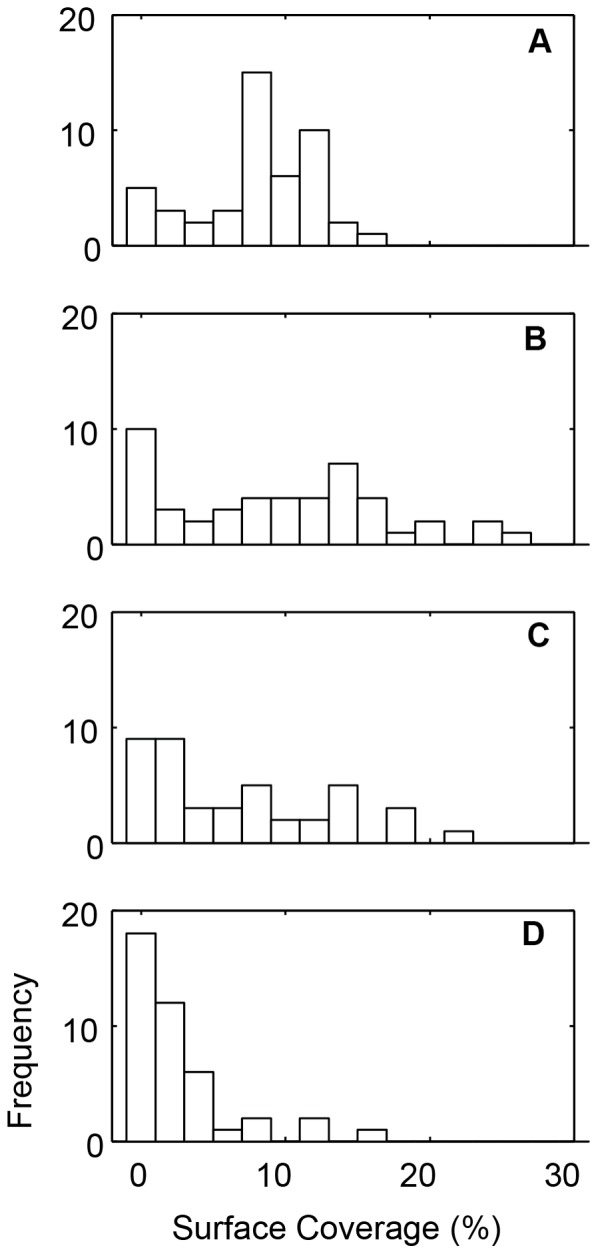
Distribution of platelet surface coverage (SC) in cohort of normal donors. Histogram of percent surface coverage (SC) for 150 s^−1^ (A), 300 s^−1^ (B), 750 s^−1^ (C), and 1500 s^−1^ (D) for the fifty normal donors.

Histograms of SC show that the data does not follow a normal distribution (Fig. 5). For each wall shear rate, there existed a group of individuals with low platelet binding. We defined low binders as the group of donors with an SC <1% for each wall shear rate, except 1500 s^−1^ because the average SC (2.7% ±0.6%) is only slightly higher than 1%. The VWF levels (58% ±16%) in the low binder groups were significantly lower (p<0.01) than the VWF levels (92% ±37%) in the rest of the cohort. There was one individual with a VWF level of 26.3 IU/dL, which would clinically be considered von Willebrand disease. This individual fell into the low binder group.

**Figure 6:**
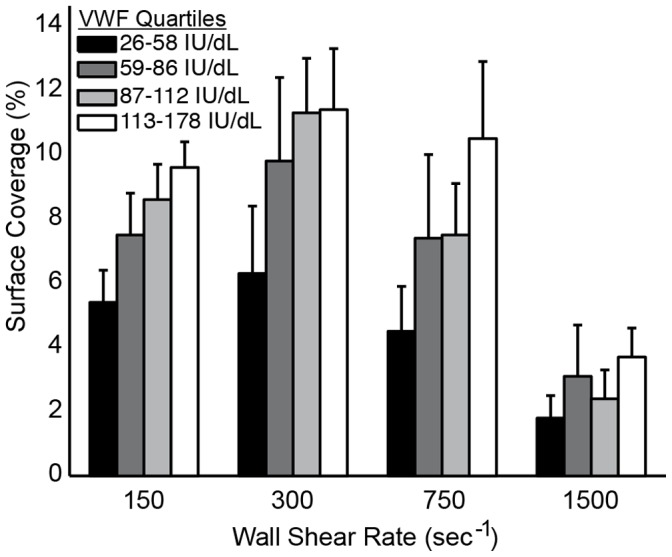
The effect of VWF plasma levels on platelet accumulation. Platelet surface coverage increases with increasing VWF plasma levels at all wall shear rates. For each shear rate, bars represent the average platelet surface coverage in each quartile of VWF levels in a cohort of normal donors (n = 50).

For the entire cohort, including the low binders, VWF levels were positively correlated with SC for all wall shear rates (Table 3). Similarly, grouping SC for each shear rate by VWF quartiles demonstrates that increasing levels of VWF lead to an increase SC (Fig. 6). The rate of platelet accumulation (V_PLT_) was also positively correlated with VWF levels at all shear rates, and negatively correlated to lag time (Lag_T_) at wall shear rates of 750 s^−1^ and 1500 s^−1^. Note that the low binders were not included in the analysis of V_PLT_ and Lag_T_ because they did not meet the criteria for calculating Lag_T_ (>1% SC). These results suggest that VWF plays a role in both platelet adhesion and aggregation in the MFA. At arterial shear rates (750 s^−1^ and 1500 s^−1^), the initial adhesion of platelets as measured by Lag_T_ decreased with increasing VWF levels. At all shear rates, V_PLT_, which account for both aggregation and adhesion, increased with increasing VWF levels. Taken together, these results suggest that VWF plasma levels are a major determinant of platelet accumulation on type 1 collagen at both venous and arterial shear rates.

**Table 3:** Spearman correlation coefficient between VWF levels and platelet surface coverage (SC), lag time (Lag_T_) and platelet accumulation velocity (V_PLT_).

Wall Shear Rate (s^−1^)	SC	Lag_T_	V_PLT_
150	0.51**	0.08	0.55**
300	0.38**	0.14	0.30*
750	0.40**	−0.42*	0.52**
1500	0.32*	−0.28	0.56*

* p<0.05;

** p<0.01.

### Platelet Accumulation and Sex

**Figure 7:**
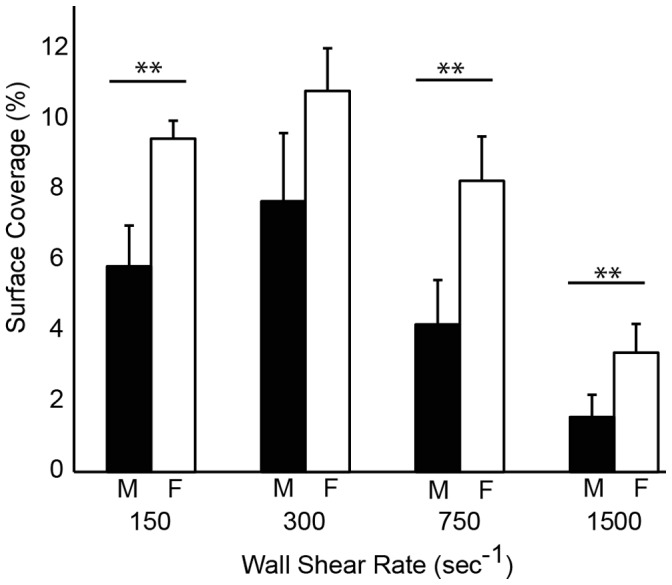
The effect of gender on platelet accumulation. Differences in platelet surface coverage (SC) between men (black bars, n = 21) and women (white bars, n = 29) at each wall shear rate. Lines with ** denotes a p<0.01 for the Mann-Whitney U-test.

Women (n = 29) had higher platelet accumulation than men (n = 21) as measured by SC at all wall shear rates (Fig. 7). However, women also had higher (p<0.05) VWF levels than men (Table 1), and thus it is not possible to decouple these variables. There was no difference between women who were (n = 6) or were not (n = 23) taking oral contraception.

### Platelet Accumulation is not Correlated to Hematocrit or Platelet Count in the Normal Range

There was a weak negative correlation between hematocrit and SC at each shear rate although this dependence was not statistically significant (data not shown). Similarly, Lag_T_ and V_PLT_ were not correlated to hematocrit. There was no correlation between platelet count on any of the metrics of platelet accumulation. The hematocrit and platelet count for every individual in the cohort was within the normal ranges (Table 1).

### Platelet Accumulation and Platelet Receptor Genotype

**Figure 8:**
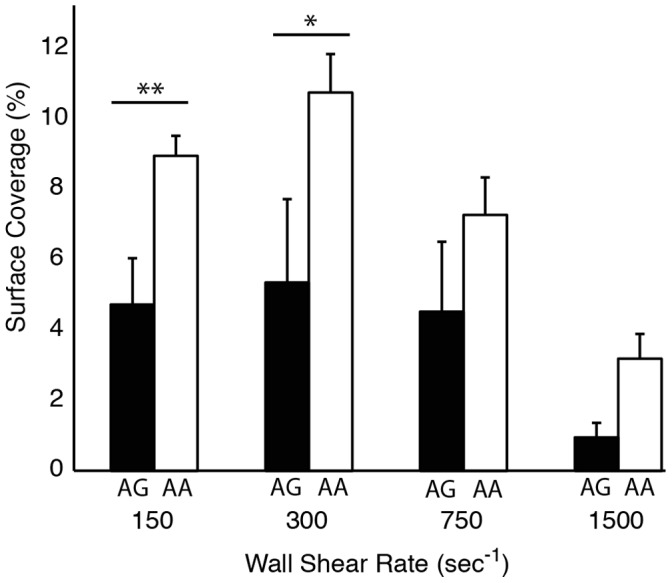
The effect of GP6 genotype on platelet accumulation. The AA genotype (white bars) yields higher accumulation than the AG genotype (black bars) at venous shear rates. Line with ** denotes a p<0.01, * denotes p<0.05 for the Mann-Whitney U-test.

In each donor we measured variants for three genes – *ITGA2, GP6* and *GP1BA* – that encode for platelet adhesion receptors – α_2_β_1_, GPVI, and GB1b – to determine if the presence of specific alleles affected platelet accumulation in the MFA. These genetic variants were selected based on their biological significance and previous reports of association with platelet or clinical bleeding and thrombotic phenotypes (Table S1). Individuals with the AA genotype of the *GP6* gene had higher SC than individuals with the AG allele (Fig. 8). Lag_T_ was not different between the two *GP6* genotypes, but V_PLT_ was significantly (p<0.05) higher for the AA genotype at 150 s^−1^. There was no difference in VWF levels between the two genotypes and no significant interaction (p = 0.64) as measured by two-way ANOVA. The frequency of the T allele of *ITGA2* in this population was 0.06 compared to the reported 0.36 in Caucasians with only four individuals in the cohort exhibiting the *ITGA2* TT genotype [23]. Consequently, there was too low an incidence to provide sufficient statistical power to measure the effect of the *ITGA2* genotype. Similarly, there was only one individual in our cohort with the more rare *GP1BA* CT genotype.

### The Effect of Sodium Citrate on Platelet Accumulation

**Figure 9:**
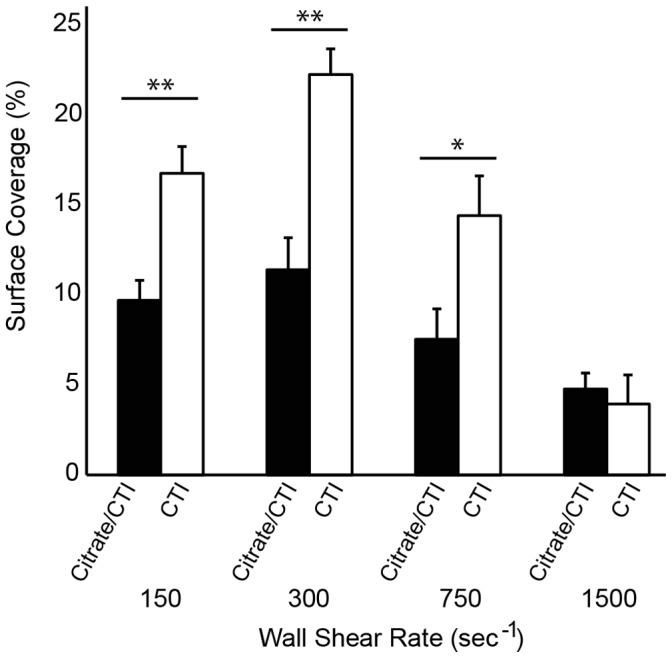
The effect of anticoagulant on platelet accumulation. For each donor (n = 10), whole blood was collected into sodium citrate and CTI or CTI only. Line with ** denotes a p<0.01 for the Mann-Whitney U-test.

We compared platelet accumulation with whole blood collected into sodium citrate and CTI and CTI alone. There was approximately a two-fold increase in SC at 150, 300, and 750 s^−1^ for CTI alone (Fig. 9). A similar SC was observed for both conditions at 1500 s^−1^. These data suggest that the effect of sodium citrate on platelet function is not completely reversible upon recalcification.

### The Lag Time for Platelet Accumulation at High Shear Rates is due to Adsorption of Plasma Proteins

**Figure 10:**
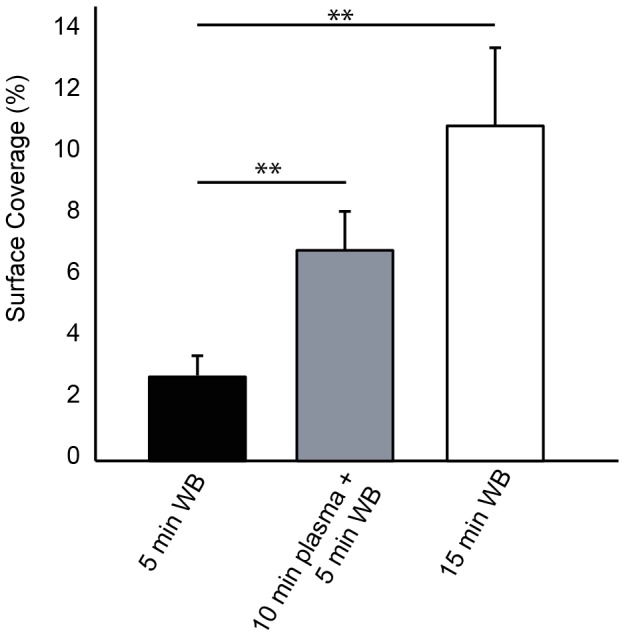
The role of plasma protein adsorption on platelet accumulation. Three conditions for each donor (n = 10) were considered; 5 min whole blood, 15 min whole blood, and 10 min plasma followed by 5 min whole blood. All conditions were performed at 1500 s^−1^. Line with ** denotes a p<0.01 for the Mann-Whitney U-test.1.

In our cohort of normal donors we found that the lag time for platelet accumulation increased with increasing wall shear rate (Fig. 4C). As a consequence, the SC at 1500 s^−1^ is significantly lower than the other wall shear rates. We hypothesized that this lag time is associated with the adsorption of plasma proteins (e.g. VWF) to the fibrillar collagen. To test this hypothesis we compared three conditions; 5 min whole blood perfusion, 10 min autologous plasma perfusion followed by 5 min whole blood, 15 min whole blood perfusion (Fig. 10). The shear rate for all conditions was 1500 s^−1^. There was a four-fold increase in SC for the 15 min whole blood perfusion compared to 5 min whole blood. There was also a significantly higher SC for a 5 min whole blood perfusion following 10 min plasma pretreatment. The lag time following the plasma pretreatment was 47.4±15.6 sec compared to 189.3±60 sec for a 5 min whole blood perfusion without the pretreatment. These data support the hypothesis that the adsorption of plasma proteins to fibrillar collagen accounts for the delayed lag time in platelet adhesion at 1500 s^−1^ compared to lower shear rates.

## Discussion

The purpose of this study was to measure the variability of platelet accumulation in MFA within the normal population and associate that variability with common factors known to effect platelet function. We examined VWF plasma levels, hematocrit, sex and platelet count as well as variants of the *GP6, ITGA2*, and *GP1BA* genes. This is the largest flow assay study of a healthy population to date. As such, it provides useful baseline data on sources of variability that must be accounted for in future studies with bleeding and thrombotic disorder patients. In addition we measured the effect of experimental conditions including collagen surface density, sodium citrate, and assay time.

The strongest determinant of platelet accumulation on type I collagen was VWF plasma levels at both venous and arterial wall shear rates. VWF levels positively correlated with total platelet area fraction, lag time, and the rate of platelet accumulation. This observation is in agreement with a previous study of platelet adhesion on type I and type III collagen at arterial shear rates in larger parallel plate flow chambers [24]. There are reports both in support of [25], [26] and against [27], [28] a role for GP1b-VWF mediated thrombus formation at venous shear rates in flow chambers. Recent studies in animal models of venous thrombosis support a role for VWF at low shear stresses [29]–[31]. The mechanism of VWF under venous flow conditions is currently unknown. Current hypotheses include a yet to be discovered low shear stress VWF receptor or interaction between VWF and DNA neutrophil extracellular traps (NETs). The VWF contribution at low shear stresses may also be related to signaling through the GP1b/V/IX complex, which is supported by reports that low levels of VWF or inhibition of GP1b/V/IX results in diminished phosphatidylserine (PS) exposure and fibrin formation at venous shear rates [32].

There was no correlation between platelet accumulation and hematocrit and platelet count within our cohort. Although these two factors have been shown to influence platelet interactions the vessel wall or adhesive substrates, their effects are limited to abnormally low levels (i.e. anemia, thrombocytopenia) [33], [34]. All of the individuals tested in this study were in the normal range of hematocrit (0.35–0.50) and platelet count (150,000–500,000/µL) and consequently, it is perhaps not surprising that there was a weak correlation with platelet accumulation. This is in agreement with other flow-based studies of platelet function where a measurable decrease in platelet accumulation was only observed for platelet counts less than 100,000/µL [34], [35].

Women had higher platelet accumulation than men in the MFA. However, women had higher VWF levels than men in our cohort, so it is not possible to decouple the two variables. There is conflicting evidence on the role of gender on platelet function. Gender differences in platelet function have been previously reported in platelet aggregometry studies with human and murine platelets [36]–[38]. Aggregation responses are higher for women in response to ADP, epinephrine, archidonic acid and collagen. However, in functional assays such as platelet spreading and platelet adhesion under flow, no gender differences were observed in mice [39].

The genetic factors we considered were alleles for the genes of three primary platelet adhesion receptors; *GP6* (GPVI), *ITGA2* (α_2_β_1_), and *GP1BA* (GP1bα). We found greater platelet accumulation in individuals with the AA genotype of the GP6 gene than the AG genotype at all four wall shear rates. There was no difference in VWF levels between the two genotypes. This observation is in agreement with a previous study showing that homozygotes for the G allele have diminished platelet accumulation on type III collagen at a shear rate of 1600 s^−1^ and longer closure times in the PFA-100 using type 1 collagen [40]. The AG genotype is also associated with an increase in an age adjusted bleeding score in individuals with type 1 von Willebrand disease [41]. The incidence of minor alleles in the ITGA2 and GP1BA gene were too small to provide adequate statistical power in this study. It is also worth noting that α_2_β_1_ density, which is determined by C807 T genotype, is correlated with platelet adhesion on type 1 collagen at 1300 s^−1^ [24]. This observation provides further evidence that collagen receptor genotype is an important factor in shear dependent platelet function.

At the time of this writing, standards have yet to be established for flow assays, although recommendations have been offered in various reports with regards to chamber size, surface coatings, blood collection, imaging, and quantification [15]–[17]. In this study, we used type 1 equine fibrillar collagen because this reagent is used in platelet aggregometry and is commonly used in flow assays for measuring platelet function. We found that adsorption from collagen solutions of 100 µg/mL or greater was a saturating condition with respect to platelet accumulation. We also found a prolonged lag time in platelet accumulation on fibrillar collagen at high arterial shear rates. Several drawbacks of using fibrillar collagens in flow assays have been previously reported, including fibers extending into the lumen of the channel [42], contamination with non-human VWF [43], and heterogeneity in fiber size and density [44]. Promising alternative approaches to fibrillar collagen include collagen related peptides [42] and collagen thin films [45]. Our group has found that collagen thin films support high levels of platelet adhesion at 1000 s^−1^ and can be patterned into micron scale features within microfluidic channels [10], [44]. The association rate of VWF adsorption to collagen thin films is over an order-of-magnitude greater than that of adsorption to fibrillar collagens [44]. As a consequence, the lag time for platelet accumulation is comparable at both venous and arterial shear rates [10].

Another outstanding issue in flow assays is image analysis [17]. We developed an image processing routine that can convert thousands of fluorescence images into platelet surface coverage in a matter of minutes. This routine can be downloaded from the Matlab File Exchange (www.mathworks.com/matlabcentral/fileexchange/) and removes some of the subjectivity in analyzing large data sets of images and greatly reduces the time needed for analysis. The signal to noise ratio is relatively low in images taking during the flow assay due to the background fluorescence of labeled platelets flowing through the field of view. Consequently, we were forced to use the conservative triangle thresholding routine [18], so as not to include background noise. Platelet surface coverage was typically 2–5% higher when calculated from images taken during the rinsing step compared to the last frame of the video using the same thresholding routine. Nevertheless, the overall trends and correlations were similar between the two types of images. One limitation of this image processing routine is that it quantifies platelet aggregate growth in two-dimensions, namely the plane of the glass slide. Hence, we are unable to draw conclusions with regards to aggregate growth in direction perpendicular to flow (height-direction), which may be the primary direction of growth following initial platelet adhesion. For example, secondary platelet activation via ADP receptor P2Y12 mainly drives aggregate growth in the direction perpendicular to flow [46].

There is evidence that sodium citrate treated blood, even when recalcified, lead to changes in the ability of platelets to adhere and support thrombin generation [47], [48]. To test the effect of sodium citrate in the MFA we conducted a side-by-side comparison of whole blood collected into sodium citrate and CTI versus CTI alone. The platelet accumulation as measured by surface coverage was roughly double with CTI alone. These data support the hypothesis that sodium citrated leads to irreversible changes in platelet function and suggest that alternative anticoagulants such as CTI are preferable for flow based platelet function assays.

We observed significantly lower levels of platelet accumulation at 1500 s^−1^ in a 5 min flow assay compared to the other shear rates. The average lag time for platelet accumulation, defined as greater than 1% SC, was 194 sec at 1500 s^−1^ compared to 43 sec at 150 s^−1^. Based on this difference it is not surprising that the end-point surface coverage for 1500 s^−1^ was quite low since these platelet aggregates only had ∼100 sec to form compare to ∼250 sec at the lower shear rates. This lag time at 1500 s^−1^ is likely due to the time required for VWF to adsorb to the collagen. This explanation is supported by data that shows platelet accumulation at 15 min was significantly higher than 5 min. In addition, collagen surfaces pre-incubated with plasma for 10 min reduced the lag time for platelet accumulation at 1500 s^−1^ from 189 sec to 47 sec. Similar reports of increased accumulation on collagen versus collagen-VWF substrates were previously reported in larger parallel plate flow assay [49].

The advantages of microfluidic platforms for platelet function assays include low blood volume requirements and the ability to design multishear high-throughput devices [8], [10]. However, there are some important limitations of microfluidic-based assays when extrapolating result to in vivo thrombus formation. Owing to the small size of microfluidic channels, they are poor models for studying the mechanisms of thrombosis in large arteries. Because the inertial forces are negligible in microfluidic channels at the shear rates typical of large arteries, there are no secondary flows, which are a hallmark of clots formed near bifurcations and stenoses. The flow is pulsatile in large arteries, whereas in most parallel plate and microfluidic flow assay studies, including this one, the flow is constant. The effect of pulsatility on platelet function is unclear as there have been few studies on the subject within flow assays. One study in an annular chamber showed there was no difference in platelet adhesion between constant and pulsatile flow in an annular chambers with 2.6 mm gap width [50], while another reported an increase in platelet activation and adhesion between constant pulsatile flows in a parallel plate flow chamber with a 100 µm gap width [51]. Another consideration is the effect of channel size on the rheological properties of whole blood. The Fahreaus effect describes the reduction of hematocrit in small channels compared to large channels. Over the normal range of hematocrit, which includes all of our subjects, the reduction in the hematocrit is ∼80% in a 50 µm channel using the relationship derived by Pries and colleagues [52]. This reduction in hematocrit leads to changes in the viscosity of the fluid, which is known as the Fahreaus-Lindqvist effect. For a hematocrit of 0.5, the reduction in the viscosity between a 1 mm channel and 50 µm channel is ∼34% [52]. Based on these consideration, the independence of platelet accumulation on hematocrit and platelet count reported here could be limited to small channels or vessels.

In summary, MFA are being increasingly used to test platelet responses under flow conditions and their potential utility in laboratory medicine is being currently explored. By carefully examining the effect of several assay dependent variables including collagen substrates, type of anticoagulation and shear rates as well as the effect of physiologic and genetic variants in a large cohort of healthy donors, we believe we have set up the first steps for larger studies that will be able to standardize these types of assay.

## Supporting Information

Table S1 **Genotypes and alleles frequencies of the three SNPs studied in the healthy control population.**
(DOC)
