# Supplementary material for: Sources of Variability in Platelet Accumulation on Type 1 Fibrillar Collagen in Microfluidic Flow Assays
Source: PLoS One. 2013 Jan 23;8(1):e54680. doi: 10.1371/journal.pone.0054680 (PMC3552855; doi:10.1371/journal.pone.0054680)
Supplement: Table S1 — Genotypes and alleles frequencies of the three SNPs studied in the healthy control population. (DOC) [file pone.0054680.s001.doc]

Table S1. Genotypes and alleles frequencies of the three SNPs studied in the healthy control population

| Gene | SNP rs # | Genotypes | | | Alleles | | Biological Significance |
| --- | --- | --- | --- | --- | --- | --- | --- |
| *GP1BA* | 6065 | CC 0.975 | CT  0.025 | TT  0.00 | C  0.988 | T  0.012 | T increased risk of cardiovascular disease, high LD with VNTR (Caucasians) [1] |
| *ITGA2* | 1126643 | CC  0.92 | CT  0.048 | TT  0.032 | C  0.944 | T  0.056 | T increased α_2_β_1_ receptor density  C decreased α_2_β_1_ receptor density [2] |
| *GP6* | 1613662 | AA  0.734 | AG  0.253 | GG  0.013 | A  0.861 | G  0.139 | G decreased platelet function and expression [3] |

SNP. Single Nucleotide Polymorphism

LD. Linkage Disequilibrium

VNTR. Variable Number of Tandem Repeats

1. Murata M, Matsubara Y, Kawano K, Zama T, Aoki N, Yoshino H, Watanabe G, Ishikawa K, Ikeda Y. Coronary artery disease and polymorphisms in a receptor mediating shear stress-dependent platelet activation. *Circulation* 1997; **96**: 3281–6.
2. Kritzik M, Savage B, Nugent DJ, Santoso S, Ruggeri ZM, Kunicki TJ. Nucleotide polymorphisms in the alpha2 gene define multiple alleles that are associated with differences in platelet alpha2 beta1 density. *Blood* 1998; **92**: 2382–8.
3. Joutsi-Korhonen L, Smethurst PA, Rankin A, Gray E, IJsseldijk M, Onley CM, Watkins NA, Williamson LM, Goodall AH, De Groot PG, Farndale RW, Ouwehand WH. The low-frequency allele of the platelet collagen signaling receptor glycoprotein VI is associated with reduced functional responses and expression. *Blood* 2003; **101**: 4372–9.
